# Experiences of LGBTQ student-athletes in college sports: A meta-ethnography

**DOI:** 10.1016/j.heliyon.2023.e16832

**Published:** 2023-06-01

**Authors:** Meng Xiang, Kim Geok Soh, Yingying Xu, Seyedali Ahrari, Noor Syamilah Zakaria

**Affiliations:** a Department of Sports Studies, Universiti Putra Malaysia, Seri Kembangan, Malaysia; b Department of Philosophy and Civilization Studies, Universiti Putra Malaysia, Seri Kembangan, Malaysia; c Department of Professional Development and Continuing Education, Universiti Putra Malaysia, Seri Kembangan, Malaysia; d Department of Counsellor Education and Counselling Psychology, Universiti Putra Malaysia, Seri Kembangan, Malaysia

**Keywords:** LGBTQ, Student-athlete, Collegiate sports, Qualitative research synthesis, Meta-ethnography

## Abstract

This study aimed to explore and describe the experiences of LGBTQ student-athletes to identify ways in which athletic staff, coaches, and others can support LGBTQ youth's safe participation in sports. Guided by the preferred reporting items for systematic reviews (PRISMA) and eMERGe reporting guidance. We conducted a meta-ethnography to synthesize qualitative research focused on student-athletes’ experiences. Fourteen studies were included in the meta-ethnography published between 1973 and 2022. Four themes were identified: (1) experiences of discrimination and violence; (2) perceived stigma; (3) internalized prejudice; and (4) coping and team support, and they were used to generate a line of argument model, which explains the stress process of LGBTQ student-athletes in the sports. LGBTQ student-athletes experience persistent discrimination in college sports, which poses a significant risk to their mental health. Meanwhile, this study identified that qualitative research on LGBTQ youth sports participation is lacking in many regions of the world and lacks knowledge of the sports participation experience of bisexual, gay, and transgender students. These findings revealed a way for research on LGBTQ-related issues and future policy and practice on LGBTQ youth-related issues in sports.

## 1 Introduction

Sport is perceived as an arena of hegemonic masculinity, perpetuating men's social dominance and the social subordination of women and gay men [[1], [2], [3], [4]]. Numerous studies have consistently demonstrated that lesbian, gay, bisexual, transgender, and queer (LGBTQ) individuals have been excluded, rejected, stereotyped, discriminated against, and treated violently in sports context [[5], [6], [7], [8], [9], [10], [11], [12]]. Given the issues faced by LGBTQ individuals in sports contexts, the International Olympic Committee identified LGBTQ athletes as the group at the highest risk for harassment and abuse in sports contexts [13]. The situation is the same in LGBTQ youth's sports participation. Many LGBTQ youth reported being the target of homophobia in sports; they feared rejection by their teammates and discrimination by coaches and officials [7]. LGBTQ youth perceived the sports context as an exclusive environment allowing blatant bullying and more subtle discriminatory behaviors [14]. As a result, LGBTQ youth were less likely to participate in sports than their heterosexual peers; In some sexual orientation groups, the gap in participation in formal sports has widened over time [15].

While a large body of quantitative research demonstrated the existence of discrimination against LGBTQ individuals in sports [16], scholars pointed out that because the inclusion criteria for LGBTQ youth in existing quantitative studies were inaccurate and quantitative studies mostly had secondary data and cannot examine the unique personal and social factors [17]. Therefore, LGBTQ-related discrimination evidence in the sports context also needs to be gathered through the voices of individual LGBTQ youth [[17], [18], [19], [20]]. There have been a considerable number of qualitative studies with LGBTQ student-athletes over the past two decades, which gathered evidence through the feelings and experiences of LGBTQ student-athletes themselves. These findings should be grouped and synthesized for ease of use. Synthesizing qualitative research evidence can lead to new policy and education practice insights, especially in sports and exercise [21]. In addition, studies are needed to apply existing evidence on LGBTQ-related sports issues and to propose, apply, and test established theories and frameworks to help people understand the experiences of LGBTQ young athletes, provide the research and practitioner community with focused and evidence-based recommendations of ways coaches, PE teachers, and sport leaders can create sport environments which are welcoming and safe for LGBTQ people. Therefore, this systematic review aimed to undertake a comprehensive meta-ethnographic synthesis of qualitative studies on LGBTQ student-athletes in college sports to generate theoretical insights that could enhance policy and practice development in this field.

Noblit and Hare [22] developed meta-ethnography, which is now widely used in education and other disciplines. Unlike other reviews that summarize results, meta-ethnography translates findings between studies to produce new insights or interpretations that were not apparent in the original research [23]. At the same time, researchers’ redevelopment and interpretation of meta-ethnography also enhanced the quality of the results of meta-ethnography [[23], [24], [25], [26]] and provided a way for the application in sports and exercise psychology [21]. Therefore, this approach could generate new evidence about the experiences of LGBTQ student-athletes in college sports. Moreover, to enhance the quality of reporting, the eMERGe reporting guidance was adopted in this meta-ethnography [27]. The eMERGe guidance has 19 components to report the findings of meta-ethnography. It requires a clear description of the method, a discussion of analytical options, and greater transparency and completeness of reporting.

In addition, the review was theoretically anchored in Meyer [28]'s minority stress theory, which has been widely applied in LGBTQ-related research in sports [[29], [30], [31]]. In the minority stress theory, environmental circumstances, minority status and identity, different stressors, and social support are all relevant to minority stress. Meyer [28] indicated that stress could affect an LGB individual's health through a distal to proximal stress process. The distal stress process is prejudicial events, mainly outside discrimination and violence against LGB individuals; the proximal stress process is expectations of rejection, concealment, and internalized homophobia. Minority stress theory can identify the causes of distal and proximal distress and guide interventions for LGBTQ-related issues at the individual and structural levels. Therefore, this study adopted minority stress theory as the theoretical framework and employed a meta-ethnographic approach to synthesize the findings on LGBTQ student-athletes' experiences in college sports.

## 2 Method

### 2.1 Study design

According to Noblit and Hare [22], meta-ethnography can be approached through seven phrases (Appendix A). Phase 1 (Selecting meta-ethnography and getting started) was explained in the introduction section. Then, based on the eMERGe reporting guidance (Appendix B), we completed the specific guidance below each phase. It is worth noting that although this review is written based on 19 components of the eMERGe reporting guidance, this meta-ethnography did not use a linear approach as meta-ethnography is an additive process [27]. The protocol for this qualitative research review was registered at INPLASY (ID: INPLASY202240041).

### 2.2 Research team and reflexivity

The first author (MX) is currently a Ph.D. student from China, and her main project is the intersection of diverse gender/sexual identities and sports. Working as a former student-athlete and now a coach in a university, the author tries to understand the experiences of LGBTQ student-athletes. The first author invited another Ph.D. student (YX) to help with the study, who collaborated with the author to conduct searching and analysis and to interpret the findings. Both authors tried to bracket existing biases or assumptions to avoid bias, using memos to record the entire synthesis process and each meeting discussion.

### 2.3 Search for relevant literature

This study systematically reviewed the relevant literature since 1973. We chose 1973 because this is the earliest time we could find for a peer-reviewed scientific study of homophobia [32]. English and Chinese databases were searched in this study, such as EBSCOhost, Scopus, PubMed, ProQuest, SAGE, and CNKI, by December 2022. Specifically, the main keywords used in the retrieval process were (a) LG, LGB, LGBT*, sexual minority, gender minority lesbian, gay, bisexuality, trans, transgender, queer, homosexuality, sexual orientation, sexual identity, gender identity, gender diversity, (b) sports, athletics, athletes, and (c) college, university. The review also supplemented the database search with a citation search of the retrieved articles and focused only on qualitative research.

### 2.4 Inclusion/exclusion criteria and study selection

**Fig. 1:**
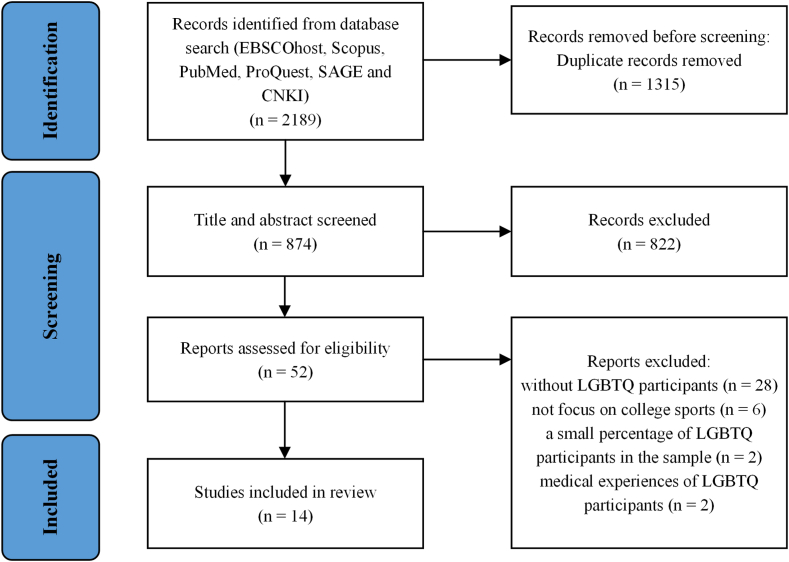
PRISMA flow diagram.

The review also met the PRISMA guidelines. After developing inclusion and exclusion criteria (Table 1), we independently searched the included databases using the search terms. The initial number of literature was 2189 articles, which resulted from limiting the years and language. After removing duplicate articles, 874 were obtained for the title and abstract screening. As many synonyms exist for LGBTQ, we initially selected studies for inclusion based on title and abstract scan rather than just reading the titles. Articles not meeting our criteria were filtered out by reading the abstracts. If the abstracts also fail to provide information, it was reserved for the full article reading. Duplicate articles were also sorted out in this stage. At this stage, we excluded many quantitative studies and theory studies. As a result, 822 articles were excluded at this stage, and 52 were considered potentially eligible. After independently reading and assessing the eligible studies, we made a joint decision that 14 studies (Fig. 1) be included in the review. It is important to explain that although there were bisexual and gay participants in two articles [33,34], the LGBTQ student-athlete representation in the sample was too low and did not meet the inclusion criteria (LGBTQ student-athlete participants must reach 80% of the total sample). Hence, we decided to exclude them after a discussion. The strict inclusion criteria were consistent with our aim for this review, as we wanted to highlight and synthesize qualitative evidence from LGBTQ students-athletes’ perspectives.

**Table 1:** Inclusion and exclusion criteria.

Inclusion criteria	Exclusion criteria
Published in a peer-reviewed academic journal.	The methodology did not use a qualitative method.
Written in English.	Was not published between 1993 and 2022.
A qualitative approach was used to collect and analyze data.	Fewer than 80% of the sample of student-athletes self-identified as LGBTQ.
Published from 1973 onwards.	Did not focus on the experiences of LGBTQ student-athletes
Participants were student-athletes who played college sports.	
Participants were self-identified as lesbian, gay, bisexual, transgender, or queer.	
LGBTQ student-athlete must reach 80% of the total sample.	
Focusing on the experiences of LGBTQ student-athletes.

### 2.5 Quality assessment

A quality assessment of all included studies was also conducted in this stage. This review used Kmet's qualitative research quality assessment checklist [35] to assess the articles' quality. This checklist includes ten key aspects of qualitative research. Research questions/objectives, design, context, theoretical framework, sampling, data collection methods, data analysis process, validation procedures, conclusions and findings, and reflexivity. Scores were used to evaluate, ranging from 0 to 2.

### 2.6 Data extraction and analysis

**Fig. 2:**
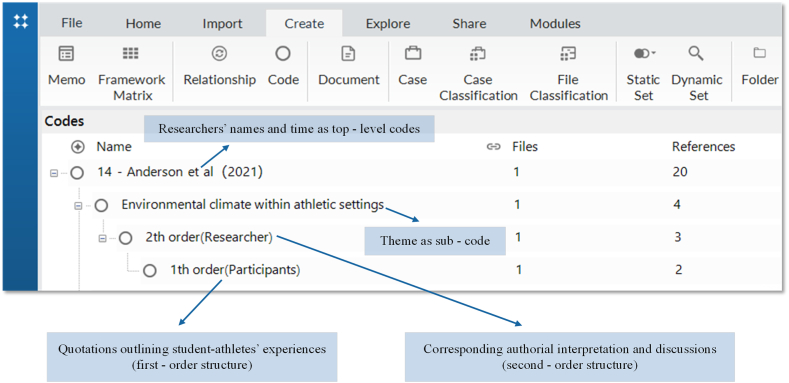
Example of data extraction using NVivo.

After we had read through the included studies, data were extracted using forms developed for the review. These included the researcher, time of publication, the focus of interest, location, participant characteristics, data collection methods, and sports. These data were extracted by the first author and checked by the second author. Like previous research [26], this study used NVivo [36] to extract the raw data from the included studies for synthesis. Included studies were uploaded to NVivo and read repeatedly, then coded each study's findings in NVivo. A hierarchical structure was used to code in NVivo, with researchers' names and time coded as top-level codes and one concept or theme from each study coded as a sub-code [25]. It made it easier for us to track the provenance of each concept or theme. Using NVivo's team function, each author's coding structure was compared, and in the process, all conflicts of opinion were resolved through discussions. We independently identified and coded quotations outlining student-athletes' experiences in college sports (first-order structure) and corresponding authorial interpretation and discussions (second-order structure) (see Fig. 2). Afterwards, these second-order constructs were further abstracted into the author's interpretation of the original researcher's interpretation (third-order constructs) [22]. Each concept or theme was then interpreted independently by both authors and recorded in the NVivo memo, and these interpretations were then compared and combined into a joint interpretation.

With Sattar et al. recommended procedure [23], we first created a list of themes that contained concepts or themes for each study and, in this way, looked to find common and recurring concepts across studies. We then reduced the themes or concepts from the different studies into relevant categories. Next, two authors independently categorized the themes. Whenever the categorization of a theme became difficult, we returned to the full text for a thorough reading and discussion until an agreement was reached. Finally, these formed categories were labeled using terms encompassing all relevant concepts. We then examined the relationships between these themes’ key concepts through juxtaposition and identifying common and recurring concepts. In addition, at this stage, we examine the background data for each study, including the context, objectives, and focus.

When the included studies span a considerable period, it is recommended that they be arranged chronologically [26]. As the studies included in this review span more than 25 years, the world has experienced a significant shift in attitudes toward LGBTQ athletes during these decades. So, both authors agreed that a chronological comparison would be appropriate. We also found that the included studies were sufficiently similar in their focus to be inter-translatable. Thus, starting with the previously created categories, we arranged each article chronologically and then compared the themes and concepts of the first article with the second article, then the combination of the former two with the third one, and so on [22]. We kept an open mind to the emerging categories in this process.

## 3 Results

### 3.1 Description of studies

Fourteen studies met the criteria and were included in the review (Fig. 1). Table 2 shows the characteristics of each included study. The results of the quality assessment are shown in Table 3. Most of the included articles were from the USA, with 12 pieces. The remaining articles were from South Africa [37] and Turkey [38]. The total sample size was 156 LGBTQ participants. Of the participants, 107 were females, 40 were males, and nine were transgender. In the subgroup of LGBTQ, 78 participants self-identified as lesbian; 51 self-identified as gay; 14 self-identified as bisexual women; nine self-identified as transgender; one self-identified dyke; one self-identified as “I don't know,” and two chose not to label their sexuality.

**Table 2:** Included studies.

No.	Paper and publish date	Country	Focus of interest	Sample	Data collection	Sport
1	Krane (1997)	USA	Homonegativism in sports described by lesbian collegiate athletes.	12 lesbians	Semi-structured interviews	Basketball, softball, golfers, soccer, track
2	Anderson (2002)	North America	Experiences of openly gay male team sport athletes	26 gay males	In-depth interviews	Bowling, cheerleading, crew, cross-country, diving, fencing, football, hockey, rodeo, rugby, soccer, speed skating, swimming, tennis, track, volleyball, water polo, wrestling
3	Stoelting (2011)	USA	intercollegiate lesbian athletes disclose their sexual identities in the sport context	16 females: 12 self-identified as lesbian; 3 self-identified as gay; and 1 self-identified as dyke	In-depth interviews	Softball, soccer, rugby, lacrosse, golf, cross country, indoor or outdoor track, field hockey, ice hockey, crew, and basketball
4	Fink et al. (2012)	USA	Perspectives of “out” lesbian or bisexual college female athletes	14 females: 11 self-identified as lesbian; 3 self-identified as bisexual	Semi-structured interviews	Softball, basketball, hockey, field hockey, volleyball, tennis
5	Melton and Cunningham (2012)	USA	experiences of lesbian athletes of color	13 females: 7 self-identified as gay; 6 self-identified as lesbian	Semi-structured interviews	Basketball and softball
6	Lucas-Carr and Krane (2012)	USA	transgender athletes experienced sport	3 transgender individuals	in-depth life history interviews	Martial art, aikido, rugby, and ice hockey
7	Mavhandu-Mudzusi (2014)	African	experiences of LGBTI students regarding sports participation	20 LGBT individuals: 5 self-identified as lesbians; 5 self-identified as gay; 5 self-identified as bisexual; 5 self-identified as transgender	In-depth interviews and field note	Not specified
8	Anderson and Bullingham (2015)	USA	experiences of openly lesbian team sports players	12 lesbians	Semi-structured interviews	Basketball, rowing, cross country, rugby, soccer, softball, track
9	Fynes and Fisher (2016)	USA	negotiation of lesbian student-athlete identities	10 lesbians	Semi-structured interviews	Track, field, rowing, volleyball, softball, basketball, soccer
10	Saraç and McCullick (2017)	Turkey	experiences of a gay male Turkish PE and sports major.	1 gay male	Semi-structured interviews	Track and field
11	Fenwick and Simpson (2017)	USA	gay male athletes experienced coming out during participation in sports.	6 gay males	Semi-structured interviews	Cross-country, football, ice hockey, lacrosse, swimming, and track
12	(Mann & Krane, 2018)	USA	college team sport climates with queer female athletes	13 females: 5 self-identified as lesbian; 3 self-identified as gay; 3 self-identified as bisexual	unstructured and semi-structured interviews	Basketball, crew, field hockey, golf, softball, track, and field
13	Klein et al. (2018)	USA	physical and athletic changes of a trans male US college athlete	1 trans male	Interviews and video diary	Cross-country and track
14	Anderson et al. (2021)	USA	experiences of female sexual minority student-athletes.	9 females: 5 self-identified as lesbian; 3 self-identified as bisexual	Semi-structured interviews	Softball, basketball, hockey, field hockey, volleyball, tennis

**Table 3:** Quality assessment of included studies.

Citation	Clear objective	Study design	Clear context	Theoretical framework	Sampling	Data collection	Data analysis	Verification and credibility	Conclusions supported by the results	Reflexivity
Krane (1997)	2	2	2	2	2	2	2	2	2	0
(Anderson, 2002)	2	2	2	2	2	2	0	0	2	1
Stoelting (2011)	2	2	2	1	2	2	2	1	2	0
Fink et al. (2012)	2	2	2	2	2	2	1	2	2	0
Melton and Cunningham (2012)	2	2	2	2	2	2	2	2	2	2
Lucas-Carr and Krane (2012)	2	2	2	2	2	2	2	2	2	1
MavhanduMudzusi (2014)	2	2	1	0	2	2	2	2	2	0
Anderson and Bullingham (2015)	2	2	2	2	2	2	1	0	2	0
Fynes and Fisher (2016)	2	2	2	2	2	2	2	2	2	2
Saraç and McCullick (2017)	2	2	2	2	2	2	2	2	2	1
Fenwick and Simpson (2017)	2	2	2	2	2	2	2	2	2	1
Mann and Krane (2018)	2	2	2	2	2	2	2	2	2	0
Klein et al. (2018)	2	2	2	1	2	2	2	0	2	0
Anderson et al. (2021)	2	2	2	2	2	2	2	2	2	1

Of the fourteen studies included in the systematic review, eight studies focused on the experiences of female athletes who self-identified as lesbian, gay, and queer [5,31,[39], [40], [41], [42], [43], [44]]; three studies focused on the experiences of gay athletes [38,45,46]; two studies focused on the experiences of only transgender athletes [47,48]; one study included all LGBTQ participants [37].

Data collection involved interviews in all but one study using a video diary [48]. While all the included articles focused on the college sports experiences of LGBTQ student-athletes, some of the studies also included a portion of the high school sports experience [45]; three studies especially focused on the “out” experiences [39,40,46]; and almost half studies recruited former student-athletes [31,39,40,42,44,47].

Participants were involved in many intercollegiate sports: basketball, softball, golf, soccer, track, crew, cheerleading, cross-country, diving, fencing, football, hockey, rodeo, rugby, speed skating, swimming, tennis, volleyball, water polo, wrestling, lacrosse, tennis. Still, these articles mentioned more than once [5,42,44] that basketball and softball were the most significant number of lesbians.

### 3.2 Description of themes

**Fig. 3:**
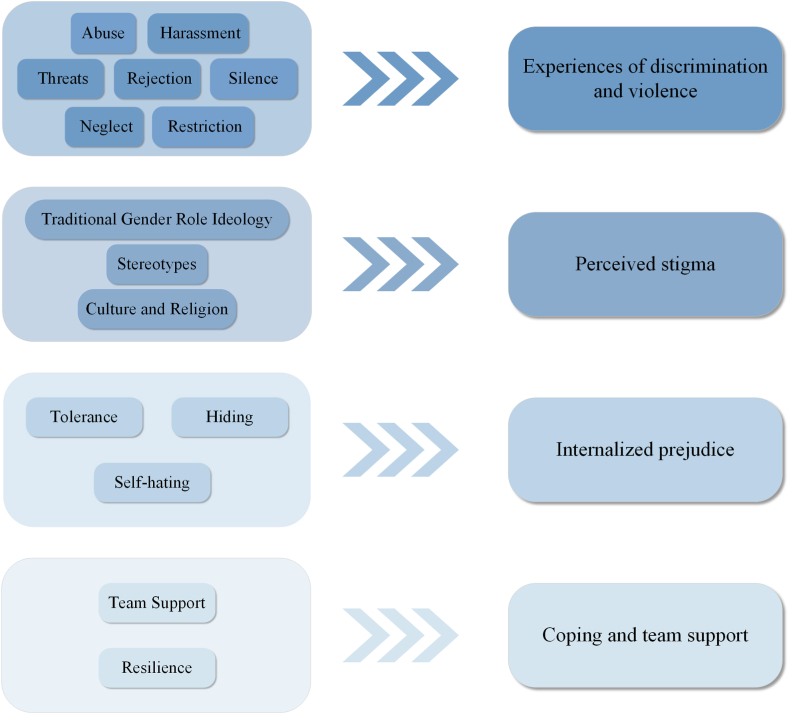
Categories abstracted to themes.

**Fig. 4:**
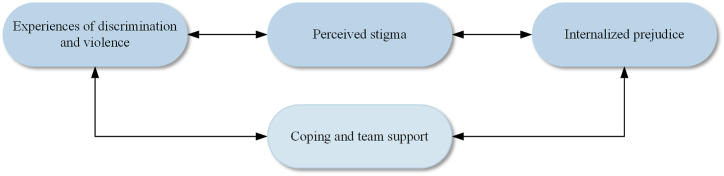
Model of the stress process of LGBTQ student-athletes in college sports.

After conducting a line of argument synthesis, four key third-order constructs were interpretively synthesized from the extracted data: (1) experiences of discrimination and violence; (2) perceived stigma; (3) internalized prejudice; and (4) coping and team support. These four themes were abstracted from 15 categories (Fig. 3). The contribution of included studies towards themes is shown in Table 4. Finally, a line of argument model was developed to express the interpretation of the results from included studies (Fig. 4).

**Table 4:** Contribution of included studies towards themes.

No.	Citation	Experiences of discrimination and violence	Perceived stigma	Internalized prejudice	Coping and team support
1	Krane (1997)	✓	✓	✓	
2	Anderson (2002)	✓		✓	✓
3	Stoelting (2011)			✓	✓
4	Fink et al. (2012)	✓	✓	✓	✓
5	Melton and Cunningham (2012)	✓	✓	✓	✓
6	Lucas-Carr and Krane (2012)	✓	✓		✓
7	Mavhandu-Mudzusi (2014)	✓			
8	Anderson and Bullingham (2015)	✓			✓
9	Fynes and Fisher (2016)	✓	✓	✓	✓
10	Saraç and McCullick (2017)	✓	✓	✓	✓
11	Fenwick and Simpson (2017)	✓	✓	✓	
12	Mann and Krane (2018)			✓	✓
13	Klein et al. (2018)	✓	✓		✓
14	Anderson et al. (2021)	✓	✓	✓	✓

#### 3.2.1 Experiences of discrimination and violence

This theme refers to external, objective stressful events and conditions that LGBTQ student-athletes face in college sports, mainly experiencing discrimination and violence, including abuse, harassment, threats, rejection, silence, neglect, and restriction. Twelve studies contributed to this theme [5,31,[37], [38], [39], [40], [41], [42], [43], [44], [45], [46], [47], [48]].

**Abuse***.* Abuse against LGBTQ Individuals made LGBTQ student-athletes feel unsafe in college sports. For example, one lesbian student-athlete described being sexually assaulted, “One of the guys picked me up and gave me the nastiest kiss on the back of my neck. I used all of my strength to fight him off, but I couldn't. He asked, ‘still Lesbian now?” [41]. In another case, one gay participant described direct violence by his teammate:

“… I was walking in the course for a race that I was expected to win, and a teammate of mine took a rock, about the size of a softball and threw it at the back of my head. … And he said, ‘what’s the big deal? It’s just that gay pussy fag kid’” [46].

This kind of abuse not only hurt the LGBTQ individual involved but was also a warning to all LGBTQ athletes. One gay student-athlete described big worries after hearing about violence against a gay man, “One of the things that was holding me back from coming out was … One of my friend's friend was beaten to a bloody pulp because they thought he was gay” [45]. Thus, both direct abuse and the afraid of being abused can leave LGBTQ student-athletes fearful and worried about their situation in the college athletic context.

***Harassment****.* The most frequent form of harassment was the use of homophobic language. LGBTQ student-athletes reported frequently hearing “fag”, “gay,” and “dyke”, as well as insulting language about gender and sexual orientation in the sports context. Homophobic language has been mentioned in many included studies. Some lesbian student-athletes thought it was verbal abuse [5], while some gay student-athletes believed it was just joking [45]. Although homophobic language could be interpreted as different motives in different contexts, most LGBTQ student-athletes indicated that it hurt their feelings and distanced them from their teammates. One lesbian student-athletes reported more severe harassment that her car was vandalized with an insulting note “die dyke” attached [41]. Both homophobic language and outright harassment seem to be common and tacitly accepted in the college sports context, and these behaviors prevent a proper understanding of the dangers of discrimination against LGBTQ student-athletes.

***Threats***. Threats usually from coaches, teammates, and athletic department staff. One coach threatened lesbian student-athletes to comply with traditional gender norms or be expulsion [5]; another coach threatened to disclose one lesbian student-athlete as a punishment, “In the meeting he told me he wasn't afraid to pull the gay card” [31]. Threats also came from teammates, causing great distress to LGBTQ student-athlete:

“… She made my life a living hell for a semester …. Somehow she found out how I didn’t want my parents to know I was gay …. She started acting like she was going to tell them … like just to hurt me or something. That would have been the worst thing ever ….” [31].

In addition, from the content of the threat we can learn that disclosure was the thing that LGBTQ student-athletes feared the most and that family was the most challenging environment for LGBTQ people to come out.

***Rejection.*** When LGBTQ status was revealed, student-athletes faced an even worse situation. LGBTQ student-athletes described plenty of experiences of being excluded from competitions and being alienated by teams [37]. One transgender athlete was criticized by her coach for not wearing a skirt, “you're not respecting yourself, you're not respecting your team” [47]. One lesbian student-athlete indicated that some team coaches refused to recruit lesbian athletes:

“I know, especially women’s basketball that it is a huge no-no. [The coach] makes it very clear to the players that will not be … that they will not be open. Moreover, that’s obviously something that’s not talked about or well known, but they’ll tell you that it’s not accepted” [40].

Likewise, some athletic department staff and coaches threatened student-athletes with having their scholarships revoked if they go to a gay bar [42]; Some lesbian student-athletes had even been expelled from the team, “He cut five people, four of them were gay” [5]. Another rejection that came with coaches was the non-recognition of LGBTQ student-athletes. For example, in Anderson and Bullingham's study [41], one coach stated, “nice girl from a ‘nice family’ couldn't dare be like that.” To make it even worse, one coach even asked a lesbian student-athlete to reject her sexual identity, “she (strength coach) hates it if you're gay, and always pushes you to go to her Bible studies … she says things like ‘God will get me’ and ‘I need to turn from my sinful ways’” [31]. For those who do not accept LGBTQ individuals, “a homosexual is not accepted as an individual. They see homosexuality as a choice” [38].

Meanwhile, teammates' rejection added to LGBTQ student-athletes’ frustration. One gay student-athlete returned to his team after a suicide attempt and was excluded by his former teammates, “Like, I was told that if I played any sports, they'd make my life living hell” [45]. In addition to being rejected by the entire team, one gay student-athlete reported lost friends due to the disclosure, “Someone had found out that we were gay and had a fit over it.…I'd say he was one of our good friends … he no longer spoke to me” [45].

***Silence.*** The silence described by LGBTQ student-athletes refers to the fact that neither teammates nor coaches normally talk about topics related to gender and sexual orientation in college sports, “nobody talks about it …. Everyone knows about everyone else, but no one talks about it …. It's not a big gay thing; you go, you dive, and you leave” [45]. One lesbian student-athlete argued that the silence had made LGBTQ-related topics taboo in college sports:

“… I feel like they talk about, you know, race, and you know, like international acceptance, the international athletes, everything but kind of gender and sexuality. You know, I feel like it’s taboo like it’s kind of not talked about” [40].

The silence was sometimes forced, and pressure came from the coaches and the athletic department, “… but it was like your coaches and the administrators who would be like, ‘just don't talk about it’” [40]. One lesbian student-athlete was even asked by their coaches to remain silent, “My coaches have these rules for what I can do and what I can't do” [41]. Silence limited LGBTQ student-athletes’ communication with teammates, coaches, and the athletic department and restricted the inclusive development of college sports.

***Neglect.*** Studies showed that student-athletes’ LGBTQ identities were neglected in favor of students’ athletic identities in the athletic department [5,31,44].

“With (coach), he (pauses) just doesn’t want to know (if a player is a lesbian). He just wants you to be a (sport) player, he doesn’t want to know about anything else, unless it makes him or the team look good” [31].

In Anderson and Bullingham's study [41], one coach neglected the needs of one lesbian student-athlete and did not offer help, which led the student to leave the team. The description of these studies showed that gender and sexual identity were still a topic that could not be openly discussed in sports teams. Most teammates and coaches chose to refuse or remain silent, which made it impossible for LGBTQ student-athletes to face up to their gender or sexual identity. They were forced to hide or even try to escape it, which made them face more psychological pressure than heterosexual athletes.

***Restriction.*** Restrictions refer primarily to the barriers to sports participation encountered by transgender student-athletes. Although transgender participation is allowed in college sports in some countries, gender expectations and rules restrict transgender athletes from participating. One transgender student-athlete described how they often experienced the embarrassment of being in a locker room that did not conform to their gender identity because they were restricted from participating in the male game:

“… we would like all change on one side of the locker room (laughing), and all the girly-girls changed on the other side of the locker room. And like we can’t touch and don’t look, but the girls on the other side of the locker room could do whatever they want” [47].

In another case, one transgender student-athlete left the sport due to a difficult process of hormone therapy [48]. Consequently, transgender student-athletes face additional challenges in college sports participation, both in the face of the restrictions of sports competition rules and the difficulties of physical transition, and the evidence of these experiences is insufficient in the literature.

Therefore, the first theme exposed various forms of discrimination and violence against LGBTQ student-athletes in college sports. The discrimination stemmed from the specific nature of the sports context as well as the social culture; however, according to LGBTQ student-athletes, educational institutions and athletic departments did not pay attention and intervene, which greatly affected the benefits that sports could bring to LGBTQ youth.

#### 3.2.2 Perceived stigma

This refers to how student-athletes perceive discrimination from the external environment and how they perceive these negative experiences. Nine studies contributed to this theme [5,31,38,40,42,44,[46], [47], [48]].

***Traditional Gender Role Ideology***. Gender role ideology is how individuals' attitudes towards the role of women and men and how this attitude is shaped by sex. In addition, gender role ideology determines the distribution of women and men in social roles in society [49]. The traditional gender role ideology required men to do the bulk of the labor while women were expected to take care of the family. In sports, corresponding to traditional gender role ideology, people expect different performances from female and male athletes. Male athletes were supposed to be aggressive, while female athletes should keep feminine. In this case, when one transgender student-athlete moved from the men's team to the women's team, they found it difficult to fit into the norms of traditional gender roles:

“I feel the big difference was that people seemed to use their bodies. Like, [for] women a lot of it is about technique. A lot of it in guys' sports, for better or worse, [is] they use their bodies almost sacrificially …. So that aggression and physicalness did not help my cause when I switched to a girl’s team” [47].

With traditional gender role ideology, female athletes must dress femininely and wear make-up and skirts [5,31,47]. One lesbian student-athlete said, “because that's just how society is. You're supposed to be straight; you're supposed to be girly” [40]. Another lesbian student-athlete also expressed frustration with the preservation of femininity in sports:

“We had to look nice because boosters were going to be there …. that meant you had to wear a dress or skirt, no exceptions. Even the straight girls didn’t like it. It’s ridiculous to have to wear a dress when it’s freezing outside (the event was in February)” [31].

Women in sports had the job of not only being athletes but also maintaining the image expected of females, which sometimes carried more weight than the role of an athlete [5]. On the other hand, Male student-athletes need to show masculinity in sports. In Saraç and McCullick's study [38], one gay student received a reprimand for acting feminine, “I was criticized because of my actions. I was told that I looked like a girl”. Influenced by traditional gender role norms, transgender student-athletes also feel the need to preserve their gender roles. Strong muscles seem to be representative of the male role, “My body is really important to me, I need to build it in a way that's going to make me feel good about myself” [48].

***Stereotypes***. Stereotypes were defined as general images or characteristics people perceive as fixed for LGBTQ athletes. First, female athletes who were masculine and successful would be put on the “lesbian” label. One lesbian student-athletes detailed how people view lesbian athletes' “butch’ appearance,” “They're super muscular. Maybe shorter haircuts … and appearance, not only their clothes but the way they walk, the way they carry their body, their posture … don't wear makeup or as much makeup as their straight teammates” [42]. In addition, lesbians were usually connected to unhealthy habits and characteristics such as drinking [42].

Labeling female athletes as lesbians divided the female team. Some female athletes distanced themselves from the lesbian label by denigrating lesbian athletes, and some lesbian athletes labeled others to avoid lesbian stigma [5]. Some sports have also been labeled as “lesbian games,” such as basketball, baseball, soccer, and rugby [5,42,44]. One lesbian student-athlete joked “… that it would actually be more shocking if a (women's) basketball player came out that they were ‘straight’” [42]. The stigma attached to women's sports was also confirmed in the South African study, “If you are a female and you play soccer or rugby, you are automatically labeled as lesbian. Whether you are straight or not, they don't care, they don't even ask” [37]. The lesbian label tried to deprive women of success through belittling and stigmatizing. At the same time, the label was discriminatory and demeaning to women, and all-female athletes suffered from the unfair treatment caused by the label.

In contrast, gay men were viewed as feminine, weak, inferior, and not good at sports. One gay student-athlete mentioned, “I think with sports …. you are supposed to be manly and then how can you be manly if you like guys. I guess because it's kind of the connotation that gay guys are feminine” [46]. “They think that homosexuals are only about being in bed; according to them, homosexuals only have sex and, then, get up” [38]. It should be noted that less evidence of stereotypes of gay athletes was found in included studies, possibly because this review only included three articles about the experiences of gay athletes.

***Culture and Religion.*** Evidence showed that culture and religion were essential in the LGBTQ student-athlete experience [38,42,44]. The study in Turkey found that a PE student's most significant barrier to self-acceptance was religion. Because homosexuality is a sin in Islam, the student described his experience of rejection by his religion:

“Of course, I rejected it at first. The reason that I didn’t accept [my homosexuality] was religion, because of religion.… I was thinking that religion always neglects homosexuality, God would not like me, and he would put me in a bad place, in the other world” [38].

In line with the influence of Islam on LGBTQ individuals, studies in the US have found that Christianity was also the cause of LGBTQ athletes' struggles with their identity. “Religiously, I don't believe that it’s right, but not everyone has the same religious values … I wish that I believed it was okay, but I don't believe that it’s okay as far as religion” [44]. Similarly, another lesbian student-athlete claimed the need to hide her identity and play another role in the Christian Athletic Association in college [42].

#### 3.2.3 Internalized prejudice

After experiencing discrimination and violence, as well as perceived sexual stigma and stereotypes in the sports context, LGBTQ student-athletes internalized these negative experiences and attitudes, developing internalized prejudices, including tolerance, hiding, and self-hating. Ten studies contributed to this theme [5,31,[38], [39], [40],[42], [43], [44], [45], [46]].

***Tolerance***. When faced with a hostile environment, some LGBTQ student-athletes choose to tolerate it [40,45]. They believed that negative comments about LGBTQ individuals were not serious. The homophobic language was considered a “joke,” “not some evil thing,” and “they didn't mean it.” Some would even join in with the “joke,” “If everybody laughed at a gay joke or something, just laughed” [5].

LGBTQ student-athletes had a high tolerance for hostile climates because they had low expectations. They anticipated that they would experience “awkward” or “weird” situations. Some even felt “surprised” if the situation exceeded expectations:

“I went out there and was kinda scared, but everyone kept being the same. You know, they kept being my friends, and there were like only two or three that stopped talking to me … and one of them, I used to be best friends with him … and as soon as he found out, he stopped talking to me” [45].

***Hiding.*** LGBTQ student-athletes often hid their gender or sexual identity when they felt unsafe in their environment [40,43]. One lesbian student-athlete declared, “I mean I never deny my sexual orientation, but I don't outwardly offer the information to people” [44]; one gay student-athlete also admitted, “When people guess it, I don't want them to make me feel that they know it” [38]. In addition, Some led segmented lives in a different context, “I think I was negotiating the representation I was putting out there of myself in each community” [42]. Some hid their sexual orientation by engaging in heterosexual topics [45] or acting heterosexual:

“I had to pretend to like girls, like, make out with girls in public and then take them back to my room and pretend to have sex with them and in reality they would want to have sex and … just trying to show people you’re not gay and you’re straight can be very exhausting. And then, make you even unhappier when you can’t be yourself and you have to fake being someone else” [46].

The hiding most came from the fear of being disclosed, “I think the biggest scare was just like the unknown. I didn't know how people would react …; so that made me fearful” [43]. Furthermore, hiding can put much psychological pressure on LGBTQ student-athletes, “It was stressful in the sense that I couldn't be honest with them …” [46]. Lying and pretending to be heterosexual led to exhaustion and frustration for LGBTQ student-athletes [39].

***Self-hating.*** LGBTQ student-athletes developed self-loathing and self-hatred for their minority identity in a hostile external climate. Some blamed the problems they encountered on their minority identity: “I immediately think that the problem comes out of my homosexuality, and that's why they behave this way” [31]. Some refused to accept the gay identity:

“I was still depressed and I was still self-hating. I still didn't want to be gay even though I knew and accepted it at that point before I came out. I didn’t. I still didn’t want to be gay at all. I was like nope, nope I know I’m gay but I don’t wart to be. I was still at that point where I was very unhappy with being gay” [46].

After being rejected by peers, one gay student-athlete said, “so that kind of made me really hate myself more” [45]. One lesbian student-athletes also expressed regret for her identity: “I'd give anything to take it all back … I don't know, maybe if I was straight life would be better. I know my life would be better” [31].

#### 3.2.4 Coping and team support

This theme refers to LGBTQ student-athletes developing resilience after coping with negative experiences with the support of their teams. Eleven studies contributed to the theme [31,[38], [39], [40], [41], [42], [43], [44], [45],^47^,^48^].

***Team*** Support. While many LGBTQ student-athletes reported many negative experiences in college sports, some described an experience of being supported by the team. For example, heterosexual student-athletes wore “gay pride socks” with their gay teammates in competition [45]; transgender athletes felt “belonged,” “awesome” [48], and “supportive” [47] in their college teams. Similarly, in women's teams, lesbian student-athletes also found support from teammates, “So I told everyone on my team … they were in the background like cheering me on, screaming, waving signs …” [40]; “they have been so supportive of me, that they even went to some gay pride events with me” [41]. Mann and Krane [43] found two types of team climates: inclusive climates and transitioning climates; among these teams, ostensibly accepting of diverse sexual orientations and introducing inclusive norms, LGBTQ students felt accepted and appreciated. One lesbian student-athlete described what it was like to be supported by the team:

“I have been in an athletic environment, and I have been on teams that have been really supportive, and that has allowed me to come out and maintain the rest of my identity, without feeling like there was something wrong with me [uh huh]. So, I mean I am just incredibly grateful for it. And you know, I have definitely been lucky in the teammates that I have had and the environments that I have been in” [39].

Notably, what could explain this kind of team support in women's team may be that lesbian was common in the women's teams [43]. For example, one lesbian student-athlete stated, “It's a community where a lot of women who play hockey are lesbians” [39]. Lesbians were common in women's teams, “I think we had at one point out of 15 or 16 girls, we had like seven gay girls … so it wasn't a big deal …” [42]; “… that it would actually be more shocking if a [women's] basketball player came out that they were ‘straight” [42].

Similarly, one lesbian student-athlete felt the situation would be better “when all your coaches are gay I guess it really doesn't matter … they were probably more understanding when they found out and probably could connect to you almost a little more than when they didn't know” [44]. A similar phenomenon was found in Carr and Krane's study [47], “And shoot, you go to a rugby party and there is a lot of androgyny happening (laughs). If you have to come out in a sport, it's a pretty safe sport to come out in”.

Therefore, when interpreting team support, the population of LGBTQ individuals needs to be considered, especially in women's teams.

***Resilience.*** Resilience was defined as LGBTQ student-athletes’ ability to adapt and cope with negative experiences. First, LGBTQ student-athletes accepted their minority identity and found it a “huge part of my life.” One lesbian student-athletes considered it a special status:

“I never really felt special. But, when I figured out I was gay it made me feel like I was finally a part of something (pauses) that I belonged. It made me feel like I was different. In a good way” [31].

Another lesbian student-athlete felt the same way, “But, you know it was just accepting myself really, and once that happened I haven't really had a problem [disclosing] since then” [39]. Once LGBTQ student-athletes accepted their identity, they would become confident, “when it is called into play you have a self-confidence about yourself that is not gendered to hold and maintain through that situation” [47] and strong, “Some things change in you … after accepting myself, I started to believe that I was more powerful. I thought that I could handle the reactions [of other people]” [38].

Therefore, the team support and personal resilience seemed to offset some of the negative effects of the external environment and positively impact the mental health of LGBTQ student-athletes.

### 3.3 Model of the stress process of LGBTQ student-athletes in college sports

The meta-ethnographic synthesis used a line-of-argument approach to generate a model of the stress process of LGBTQ student-athletes in college sports (Fig. 4). The model illustrates four main dimensions of the experiences of LGBTQ student-athletes. First, LGBTQ student-athletes experience constant discrimination and violence in the college sports environment, often from teammates and coaches; abuse, harassment, threats, rejection, silence, neglect, and restriction are the primary manifestations of discrimination. Meanwhile, LGBTQ student-athletes perceive stigma in the sports context, including traditional gender roles, stereotypes of LGBTQ individuals, and pressures from cultural and religious, which make LGBTQ student-athletes vigilant about their environment. As a result, high levels of stigma can lead to chronic pressure, which leads LGBTQ student-athletes to endure hostility from the external environment, hide their identity, and even self-hating. Under certain conditions, such as receiving team support, some LGBTQ student-athletes would develop resilience and become confident and strong. Each dimension is interrelated, and ultimately, these experiences can have different health effects on LGBTQ student-athletes. It is worth noting that this is a dynamic process: People's attitudes towards LGBTQ athletes evolve with the culture and the climate of the college sports context changes with the renewal of teammates or coaches. More importantly, LGBTQ student-athletes' acceptance of their minority status also influenced the perceptions of their identity.

## 4 Discussion

This study used a meta-ethnographic approach to synthesize qualitative studies that focused on the experiences of LGBTQ student-athletes in college sports. We conducted this review to identify ways in which athletic staff, coaches, and others can support this population to participate safely in sports.

The results show that most of the studies are from the United States and therefore lacked evidence of the experiences of LGBTQ student-athletes from other regions. The results also show a dominance of research on lesbians and a lack of research on gay, bisexual, and trans youth. Therefore, there are considerable gaps in the literature, and scholars need more effort to increase knowledge in this area across different regions and LGBTQ subgroups.

The results of this meta-ethnography are generally consistent with the results of the quantitative study [14,[50], [51], [52], [53], [54], [55]]; there is solid qualitative evidence that LGBTQ youth are discriminated against in sports. Especially gay student-athletes have been found to experience even more egregious acts of discrimination accompanied by physical abuse. Roper and Halloran [56] found that male student-athletes have a more negative attitude towards lesbians and gay men than female student-athletes. Compared to women's teams, male teams had a less tolerant climate, and gay male athletes were more worried about being alienated from their teams [57]. As a result, sexual minority men were less likely to engage in physical activity or participate in team sports than heterosexual men [58]. In addition, the qualitative evidence for transgender youth in this study is consistent with the review of sport and transgender people [59], which found that the sport's policies impose many restrictions that affect transgender youth's sports participation opportunities and benefits.

Evidence of homophobic language continues to require attention. Scholars have found frequent homophobic language was used in youth team sports [60], and homophobic language was an important tool for stigmatizing gays and lesbians in sports [55]. Homophobic language can maintain heteronormativity in sports, and the frequent use of homophobic language contributes to the perception of gay male identity as an inferior form of masculinity, marginalizing all non-heterosexuals individuals in sports [45]. While scholars have called for the use of interventions to reduce the occurrence of homophobic language in sports [60], a recent study of a social cognitive education intervention in youth rugby teams found that the intervention did not significantly reduce the use of homophobic language or change related norms and attitudes [61]. Therefore, research on homophobic language needs to continue, and scholars need to explore more effective ways to intervene in the appearance of homophobic language in sports contexts.

The results show some positive experiences for LGBTQ student-athletes, such as team support. However, we must interpret this result cautiously because the results also revealed a significant number of LGBTQ teammates in team-supported settings [39,42,43,47]. Therefore, these positive experiences may not indicate that the sports environment is inclusive of LGBTQ individuals. This support and acceptance may come from LGBTQ teammates. On the other hand, due to the presence of numerous LGBTQ athletes on the team, heterosexual students will have more opportunities to engage with LGBTQ students, therefore enhancing the communication generated between the two groups. Roper and Halloran [56] found that student-athletes who reported having contact with gay men or lesbians had significantly more positive attitudes toward gay men or lesbians; Pariera et al. also inferred that greater exposure to LGBTQ athletes might help reduce negative assumptions held by heterosexuals [62]. In conclusion, future research needs to pay attention to the number of LGBTQ individuals on the team when examining the climate of inclusive teams, as this is a key factor in interpreting the results.

Furthermore, the results of this review are primarily consistent with the minority stress theory [28]. LGBTQ student-athletes are continually exposed to discrimination and violence in the sport context; after perceiving identity stigma, LGBTQ student-athletes would develop internalized prejudices, which affect their mental health. Although team support can reduce mental health risks for LGBTQ student-athletes, there is insufficient evidence that the sport context is inclusive of LGBTQ individuals in this study. In addition, consistent with previous studies [63,64], this meta-ethnography found that resilience was a key factor affecting the health of LGBTQ student-athletes. However, the mechanism by which this resilience is generated in a sports context is unclear, and further research is needed to explore this area. Moreover, Given the important positive role of recovery factors for LGBTQ individuals, future relevant research could explore the experiences and mental health effects of LGBTQ individuals using combined minority theory and other identity theories, such as the homosexual lifespan development model [65].

This study highlights the importance of interventions for LGBTQ-related issues in sports participation. The development of prevention and interventions needs to include the interrelationship of the four components of advocacy, policy, education, and research [66]. Therefore, educational institutions should incorporate inclusion into relevant curricula and work to increase campus-wide dialogue on LGBTQ-related topics [67]. Educational institutions and athletic departments should incorporate policies and procedures, such as “prejudice response team” orientation [68], to ensure a safe and affirming environment for LGBTQ student-athletes. Provide professional development for athletic teachers and coaches, increasing their awareness and knowledge of the importance of sensitive language in sports [69]. Meanwhile, given that LGBTQ-related education resources cannot be proven effective in sports contexts at this time, educational resources need to be assessed to ensure effectiveness [70]. Lastly, to advance the field, it is necessary for researchers and funding agencies to conduct research, for example, using minority stress theory and other related theories to study LGBTQ youth-related issues in different sports contexts and regions.

## 5 Implication and limitation

Based on existing knowledge about LGBTQ individuals in sports participation, this study synthesized qualitative research which explores the experiences of LGBTQ youth in college sports. In addition, building on the literature on minority stress theory, this study establishes the stress process model for LGBTQ student-athletes, emphasizing the importance of educational institutions and athletic departments understanding and intervening in LGBTQ-related issues. We must continue our theoretical and conceptual exploration of the experiences and mental health-related issues of LGBTQ individuals. A more comprehensive understanding of the LGBTQ youth's experience allows us to fully develop policies and practices that protect the safe sport participation of LGBTQ youth.

This study also has some limitations. The variation in the literature and resources on LGBTQ student-athletes was widespread. LGBTQ and sexual minorities were utilized for the criteria of search, which could remove a few articles that used the terminologies such as non-cisgender and non-binary. In addition, this review included only qualitative studies and may have lost a small portion of the evidence for LGBTQ student-athletes in mixed studies.

## 6 Conclusion

This study used meta-ethnography to synthesize the experiences of LGBTQ student-athletes in college sports. We hope this study provides a valuable overview of qualitative evidence that can serve as a foundation to support inclusive and diverse policies and practices in educational institutions and athletic departments. Although more scholars are focusing on issues related to LGBTQ youth in sports contexts, these studies are unevenly developed, and there are many regions in the world where LGBTQ youth are discriminated against in sports contexts due to cultural, religious, and other factors, and we do not have a clear understanding of their actual situation. Therefore, we call on more regional scholars to engage in this field and work together to build a safe and inclusive sports context for LGBTQ youth.

## Author contributions

Conceptualization, MX.; methodology, MX; data collection, MX and YX.; data analysis, MX and YX; data curation, MX; writing—original draft preparation, MX; writing—review and editing, MX, YX, and SA; supervision, KS, SA, and NZ. All authors have read and agreed to the published version of the manuscript.

## Data availability statement

Data included in article/supplementary material/referenced in article.

## Additional information

Supplementary content related to this article has been published online at [URL].

## Declaration of competing interest

The authors declare that they have no known competing financial interests or personal relationships that could have appeared to influence the work reported in this paper.
