# Supplementary material for: Experiences of LGBTQ student-athletes in college sports: A meta-ethnography
Source: Heliyon. 2023 Jun 1;9(6):e16832. doi: 10.1016/j.heliyon.2023.e16832 (PMC10275786; doi:10.1016/j.heliyon.2023.e16832)
Supplement: Multimedia component 1 [file mmc1.docx]

Appendix A. Seven phrase of meta-ethnography (Noblit & Hare, 1988)

Phase 1—Selecting meta-ethnography and getting started

Phase 2—Deciding what is relevant Methods

Phase 3—Reading included studies

Phase 4—Determining how studies are related

Phase 5—Translating studies into one another

Phase 6—Synthesizing translations

Phase 7—Expressing the synthesis
