# Supplementary material for: Experiences of LGBTQ student-athletes in college sports: A meta-ethnography
Source: Heliyon. 2023 Jun 1;9(6):e16832. doi: 10.1016/j.heliyon.2023.e16832 (PMC10275786; doi:10.1016/j.heliyon.2023.e16832)
Supplement: Multimedia component 2 [file mmc2.docx]

# The eMERGe meta-ethnography reporting guidance

| **No.** | **Criteria headings** | **Reporting criteria** | **Location where item is reported** |
| --- | --- | --- | --- |
| **Phase 1—Selecting Meta-ethnography and getting started** | | | |
| **Introduction** | | | |
| **1** | Rationale and context or the meta-ethnography | Describe the gap in research or knowledge to be filled by the meta-ethnography and the wider context of the meta-ethnography | Page 3,  Line 80-96 |
| **2** | Aim(s) of the meta-ethnography | Describe the meta-ethnography aim(s) | Page 4,  Line 97-100 |
| **3** | Focus of the meta-ethnography | Describe the meta-ethnography review question(s) (or objectives) | Page 4,  Line 124-126 |
| **4** | Rationale for using meta-ethnography | Explain why meta-ethnography was considered the most appropriate qualitative synthesis methodology | Page 4,  Line 101-109 |
| **Phase 2—Deciding what is relevant** | | | |
| **Methods** | | | |
| **5** | Search strategy | Describe the rationale for the literature search strategy | Page 5,  Line 146-156 |
| **6** | Search processes | Describe how the literature searching was carried out and by whom | Page 6,  Line 159-160  Figure 1 |
| **7** | Selecting primary studies | Describe the process of study screening and selection, and who was involved | Page 6,  Line 160-170  Table 1 |
| **Findings** | | | |
| **8** | Outcome of study selection | Describe the results of study searches and screening | Page 6,  Line 158-176  Figure 1 |
| **Phase 3—Reading included studies** | | | |
| **Methods** | | | |
| **9** | Reading and data extraction approach | Describe the reading and data extraction method and processes | Page 6-7,  Line 185-203  Figure 2 |
| **Findings** | | | |
| **10** | Presenting characteristics  of included studies | Describe characteristics of the included studies | Table 2 |
| **Phase 4—Determining how studies are related** | | | |
| **Methods** | | | |
| **11** | Process for determining how studies are related | Describe the methods and processes for determining how the included studies are related:  - Which aspects of studies were compared  AND  - How the studies were compared | Page 8,  Line 227-251 |
| **Findings** | | | |
| **12** | Outcome of relating studies | Describe how studies relate to each other | Page 9,  Line 253-254  Table 4 |
| **Phase 5—Translating studies into one another** | | | |
| **Methods** | | | |
| **13** | Process of translating studies | Describe the methods of translation:  - Describe steps taken to preserve the context and meaning of the relationships between concepts within and across studies- Describe how the reciprocal and refutational translations were conducted- Describe how potential alternative interpretations or explanations were considered in the translations | Page 7,  Line 204-214 |
| **Findings** | | | |
| **14** | Outcome of translation | Describe the interpretive findings of the translation. | Page 9-19,  Line 260-582 |
| **Phase 6—Synthesizing translations** | | | |
| **Methods** | | | |
| **15** | Synthesis process | Describe the methods used to develop overarching concepts (“synthesised translations”)  Describe how potential alternative interpretations or explanations were considered in the synthesis | Page 7-8,  Line 215-224  Figure 3 |
| **Findings** | | | |
| **16** | Outcome of synthesis process | Describe the new theory, conceptual framework, model, configuration or interpretation of data developed from the synthesis | Page 9,  Line 253-259  Page 19,  Line 583-603  Figure 4 |
| **Phase 7—Expressing the synthesis** | | | |
| **Discussion** | | | |
| **17** | Summary of findings | Summarize the main interpretive findings of the translation and synthesis and compare them to existing literature | Page 19-21,  Line 605-669 |
| **18** | Strengths, limitations, and reflexivity | Reflect on and describe the strengths and  limitations of the synthesis:  - Methodological aspects—for example, describe how the synthesis findings were influenced by the nature of the included studies and how the meta-ethnography was conducted.  - Reflexivity—for example, the impact of the research team on the synthesis findings | Page 22,  Line 686-701  Page 5,  Line 137-145 |
| **19** | Recommendations and conclusions | Describe the implications of the synthesis | Page 22-23,  Line 703-712 |

France, E.F., et al., *Improving reporting of meta-ethnography: the eMERGe reporting guidance.* BMC Medical Research Methodology, 2019. **19**(1): p. 25.
